# Supplementary material for: SARS-CoV-2 Spatiotemporal Genomic and Molecular Analysis of the First Wave of the COVID-19 Pandemic in Macaé, the Brazilian Capital of Oil
Source: Int J Mol Sci. 2022 Sep 29;23(19):11497. doi: 10.3390/ijms231911497 (PMC9569756; doi:10.3390/ijms231911497)
Supplement: Supplementary file 1 [file ijms-23-11497-s001.zip › ijms-1898143-supplementary.pdf]

Supplementary material

Table of content

Table S1 List of detected mutations from 96 sequences of SARS-CoV-2 from Macaé 2

Figure S1 Clinical data from Macaé patients with SARS-CoV-2 sequenced samples......... 3

Figure S2 Non-nucleocapsid mutations are correlated with false-negative diagnosis from rapid antigen tests 4

Figure S3 Ramachandran plot from SARS-CoV-2 modeled spike protein..........................5

Genome Region	Amino Acid substitution
ORF1	P314L, D1228N, C3790F, K634N, E754K, V1019D, V1493L, T1055I, T267M, V3917G, N704D, A1352V, P80S, H2388Y, A2222S, L251W, K798T, V3968F, S944L, M592I, M2025I, I1411V, R2875H, T882I, M897I, D2361A, D51Y, V195I, R3323C, A2168V, S2430G, T2432I, V1629F, T2537I, R1729C, C1744Y, L3930F, A2618T, A2142V, K2557R, P255S, A2431V, A1151V, E1293A, A86V, G912S, D1585N, A591T, V1570A, P1223L, S610L, P892S, L3606F, P1975S, D582N, D582N, T284I, P970L, D1585N, T3058I, D589N, A34S, A1884V, A647S, I1468V
ORF3	T14I, S60A, G100C, Q57H, V256F, L101F, G174C, G49V, D155Y, H78Y
ORF6	I33T
ORF7	P68T, C15F, L96F, L12F, V93F, A55T
ORF8	Q72H, P36S, S82P, T87I
ORF9	D2N, T95M, S6I
ORF10	D31H
ORF14	G50R, G50E, V40F, E51*
S	D614G, R78M, S494P, V1176F, T29I, D1168Y, S704A, G1035V, P631S, V642F, D574Y, V1122L, T678I, E324G, V642F, V1176L, V1264L, V308L, Q23K, V70F, G842V
N	R203K, G204R, I292T, A398V, G5E, S193I, D377Y, Q9H, G236C,
M	A98S

Table S1. List of detected mutations from 96 sequences of SARS-CoV-2 from Macaé

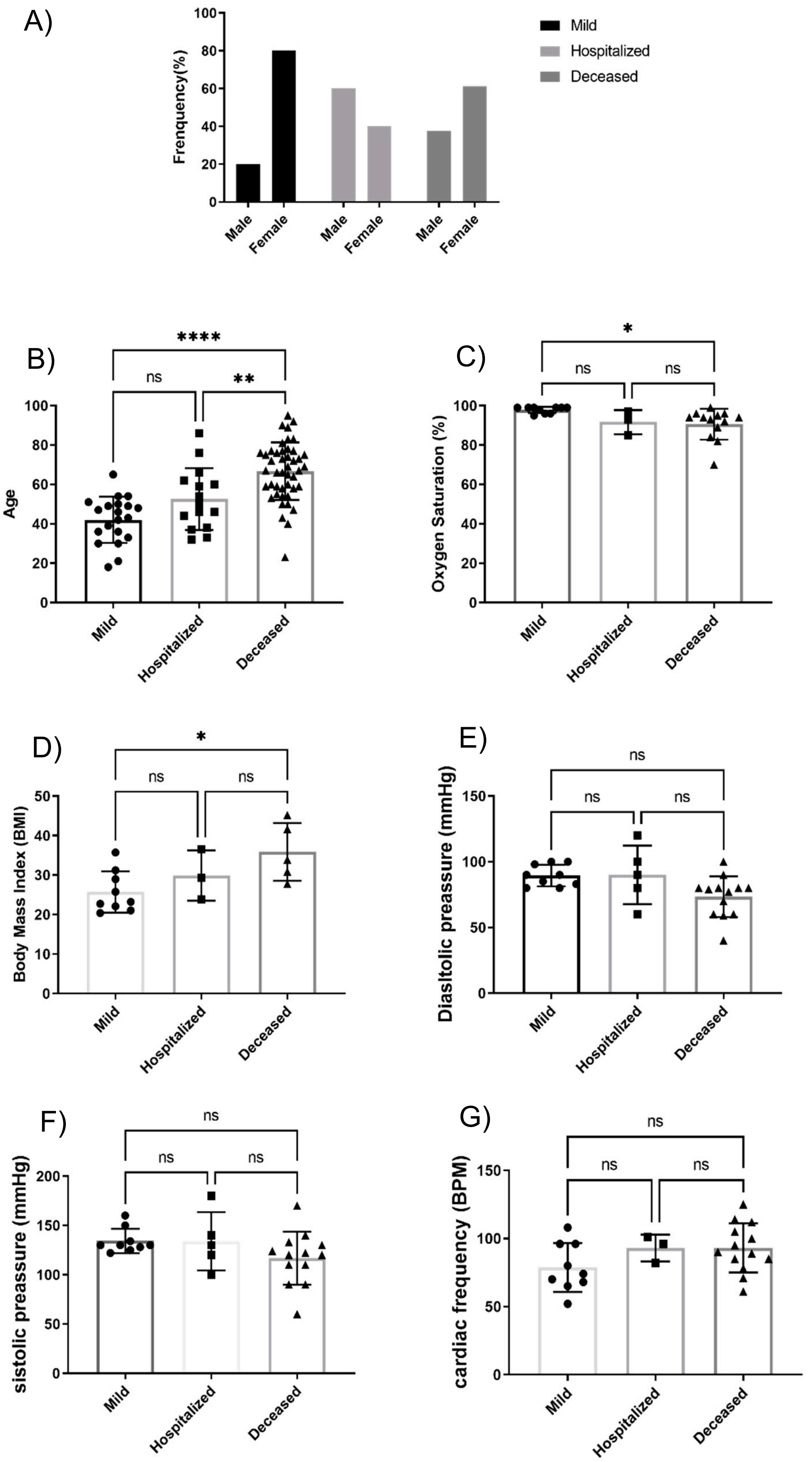

Figure S1. Clinical data from Macaé patients with SARS-CoV-2 sequenced samples. A-G) Histogram of clinical variables from patients with different levels of severity. * p< 0,05, ** p< 0,01, **** p< 0,0001.

A) B)

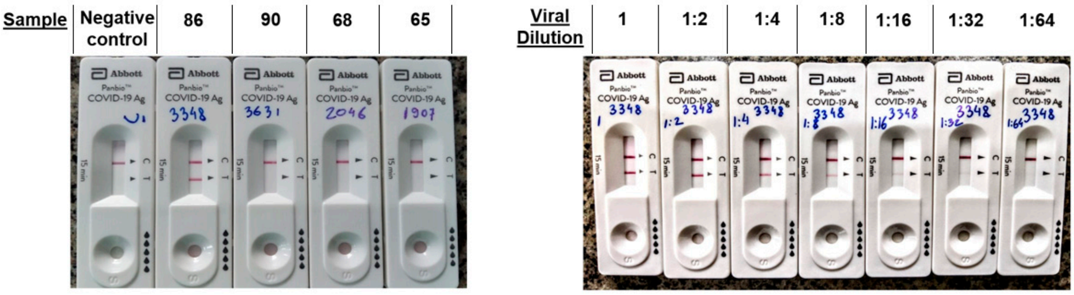

C)

	Ct			
SAMPLE	N1	N2	RNAseP	Mutations
86	18,2	20,49	23,96	ORF1a:L3606F,ORF1b:P314L,S:D614G,ORF6:I33T,N:R203K,ORF14:G50R,ORF14:G50E,N:G204R,N:I292T
90	21,34	23,31	27,43	ORF1a:D589N,ORF1b:A34S,ORF1b:P314L,ORF1b:A1884V,S:Q23K,S:D614G,ORF3a:H78Y,ORF6:I33T,N:R203K,ORF14:G50R,ORF14:G50E,N:G204R,N:I292T
68	23,77	23,73	25,93	ORF1b:P314L,S:D614G,S:V1122L,ORF6:I33T,N:R203K,ORF14:G50R,ORF14:G50E,N:G204R,N:I292T
65	23,93	26,5	31,15	ORF1b:P314L,ORF1b:K2557R,S:D614G,S:P631S,S:G1035V,ORF3a:Q57H,ORF6:I33T,N:R203K,ORF14:G50R,ORF14:G50E,N:G204R,N:I292T

Figure S2 Non-nucleocapsid mutations are correlated with false-negative diagnosis from rapid antigen tests. A) Different SARS-CoV-2 sequenced samples were chosen by the mutation profile for rapid antigen test detection. B) The positive sample detected on rapid antigen test was serial diluted to analyze the test sensibility in aspects of viral concentration. C) Samples description of cycles threshold (Ct) from RT-PCR detection, and list of mutations found from genome sequencing.

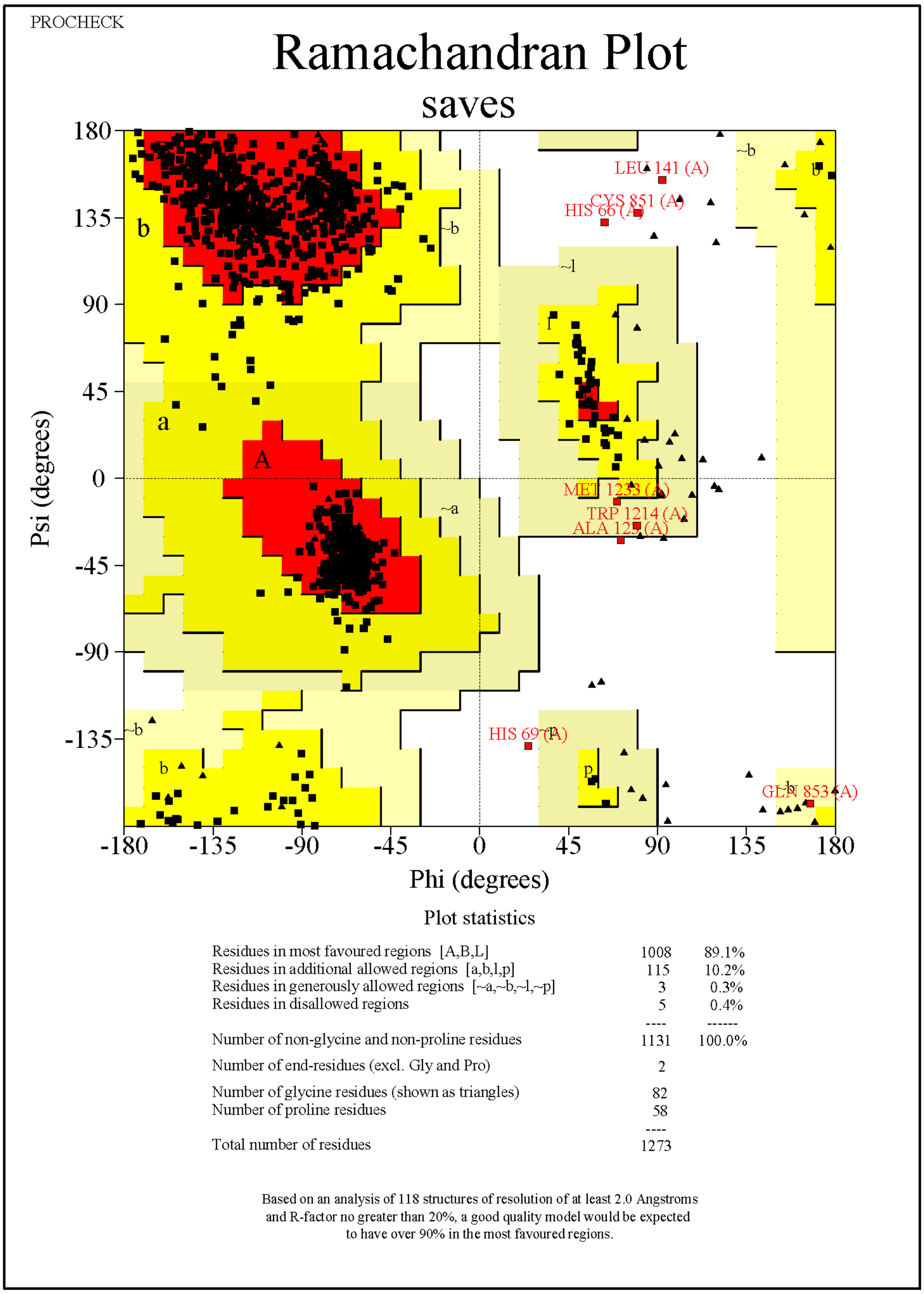

Figure S3. Ramachandran plot from SARS-CoV-2 modeled spike protein.
